# Effectiveness of adjuvant chemotherapy for elderly patients with lymph node-positive colorectal cancer

**DOI:** 10.1186/s12957-016-0959-5

**Published:** 2016-07-28

**Authors:** Tetsuro Tominaga, Takashi Nonaka, Yorihisa Sumida, Shigekazu Hidaka, Terumitsu Sawai, Takeshi Nagayasu

**Affiliations:** 1 Department of Surgical Oncology, Nagasaki University Graduate School of Biomedical Science, 1-7-1 Sakamoto, Nagasaki, 852-8501 Japan; 2 Department of Cardiopulmonary Rehabilitation Science, Nagasaki University Graduate School of Biomedical Science, 1-7-1 Sakamoto, Nagasaki, 852-8501 Japan

**Keywords:** Adjuvant chemotherapy, Colorectal cancer, Elderly patients

## Abstract

**Background:**

Several guidelines state that postoperative adjuvant chemotherapy (AC) confers survival benefits to patients with lymph node-positive colorectal cancer. However, older patients are usually not administered AC due to the higher risk of side effects. The aim of this study was to evaluate the benefit of AC for elderly patients (EP) and examine its tolerability.

**Methods:**

Data from 204 patients with lymph node-positive colon cancer were retrospectively analyzed. Patients were subdivided into two groups: EP, >75 years old (*n* = 53) and young patients (YP), <75 years old (*n* = 151). Clinicopathological features, type of chemotherapy, and outcomes were compared between groups.

**Results:**

Frequency of comorbidities and performance status were significantly higher in EP (*p* < 0.01 each), a greater proportion of YP (76 %) than EP received AC (40 %, *p* < 0.01), and YP received combination therapy more frequently than EP (*p* < 0.01). In terms of side effects, few EP showed severe side effects. Both YP and EP gained survival benefits from AC (*p* = 0.07 and *p* < 0.01, respectively).

**Conclusions:**

AC should not be withheld from eligible EP purely because of age.

**Electronic supplementary material:**

The online version of this article (doi:10.1186/s12957-016-0959-5) contains supplementary material, which is available to authorized users.

## Background

Colorectal cancer is one of the most common cancers worldwide, with an incidence of 1.2 million per year globally [1]. Generally, as the population ages, the incidence of elderly colorectal cancer patients is likely to increase.

According to the tumor-node-metastasis system, colorectal cancer with lymph node metastasis is defined as stage III disease [2]. About 50 % of patients with stage III cancer reportedly experience disease recurrence, such as local recurrence or distant metastasis, and the 5-year survival rate is 68–77 % [3, 4]. In the 1990s, the concept of adjuvant chemotherapy (AC) after curative resection for patients with stage III colon cancer was established to improve long-term outcomes [5]. AC can reportedly result in a 30 % decrease in relapse rates compared with surgery alone. Furthermore, several randomized controlled studies have revealed that stage III colon cancer patients benefit in terms of both relapse-free survival and overall survival using combination therapies that include oxaliplatin [6, 7].

However, not all patients with stage III colon cancer receive postoperative AC, with such treatment withheld from 48 to 77 % of colon cancer cases [8–10]. In general, elderly patients (EP) display higher rates of comorbidities, including cardiovascular disease, diabetes mellitus, and pulmonary disease, than younger patients (YP) [11–13]. As EP may experience a higher rate of side effects compared to YP, the decision to start postoperative AC for EP should be made carefully [9]. The Adjuvant Colon Cancer End Points (ACCENT) group assembled patient data from 18 trials testing fluoropyrimidine-based AC for patients with stage II or III colorectal cancer. Previous analyses of data from ACCENT comparing surgery alone with surgery followed by fluorouracil-based AC have revealed that patients ≥70 years old experienced similar benefits from AC compared with YP [14, 15]. However, another study revealed that EP seemed to gain reduced benefit from the addition of oxaliplatin to fluoropyrimidine in the adjuvant setting.

The aim of this study was to evaluate the effectiveness of AC for EP with colorectal cancer. We also examined the features of selected chemotherapeutic agents and their tolerability.

## Methods

### Patients

The institutional review board approved this retrospective observational study. Informed consent was obtained from all patients prior to surgery.

From January 2005 to December 2014, a total of 790 colorectal cancer patients underwent colorectal resection of the primary cancer in the Department of Surgical Oncology at Nagasaki University Graduate School of Biological Sciences. Among these, 215 patients were diagnosed with lymph node-positive colon cancer based on histopathological examination. Although neoadjuvant chemotherapy (NAC) is usually administered to patients with locally advanced colorectal cancer, the present study excluded 11 patients who received NAC to avoid confounding effects on AC. As a result, data were obtained for a final total of 204 patients with lymph node-positive colon cancer. These patients were subdivided into two groups: EP, ≥75 years old (*n* = 53) and YP, <75 years old (*n* = 151).

Before surgery, the appropriateness of resection was determined by abdominal CT and colonoscopy. The following data were retrospectively collected: age, sex, performance status, tumor markers including CEA and CA19-9, International Union Against Cancer tumor stage, operation time, blood loss, and postoperative data (including pathology, lymphatic and vessel invasion, depth of tumor invasion, hospital stay, and 30-day morbidity and mortality rates). Postoperative complications were graded according to the Clavien-Dindo classification categorizing surgical complications from grades 1 to 5, based on the invasiveness of the treatment required. In the present study, complications were defined as conditions that required treatment (Clavien-Dindo classification grades 2–5).

Colectomy, anterior resection, and abdominoperineal resection plus lymph node resection were performed according to the guidelines of the Japanese Society for Cancer of the Colon and Rectum. Either hand-sewn anastomosis or end-to-end anastomosis using a double-stapling technique was performed, depending on tumor location. Mortality and morbidity data were collected from the databases of our department and collaborating hospitals.

### AC

AC was started within 4–8 weeks after surgery, using 5-fluorouracil, TS-1, or capecitabine as a single-agent chemotherapy or oxaliplatin, 5-fluorouracil, and folinic acid (FOLFOX), S-1 and oxaliplatin (SOX), or capecitabine and oxaliplatin (XELOX) as a combination therapy. The side effects were graded according to Common Terminology Criteria for Adverse Events version 4.0.

### Statistical analysis

Data from the different groups were compared using Student’s *t* test. Continuous data are expressed as mean ± standard deviation (SD). On univariate analysis, comparisons of categorical variables were performed using the chi-square test or Fisher’s exact test. Values of *p* < 0.05 were considered significant. Overall and disease-free survival rates were calculated according to Kaplan-Meier methods. Differences between groups were tested for significance using the log-rank test. All statistical analyses were performed using SPSS version 22 software (SPSS, Chicago, IL).

## Results

### Clinicopathological features and parameters

Table 1 shows the characteristics of each group. Sex, age, body mass index, tumor location, tumor type, tumor size, lymph node metastasis, and concentrations of tumor markers, including CEA and CA19-9, did not differ significantly between groups. Frequency of comorbidities and performance status were significantly higher in EP than in YP (*p* < 0.01 each). A number of patients who had hypertension, cardiac disease, or brain infarction were identified among EP.

**Table 1:** Relationship between patient age and clinicopathological features

	Age <75 years	Age ≥75 years	*p*
*n*	151	53	
Age, years	61 (30–74)	81 (75–94)	
Sex (male/female)	85 (56.3 %)/66 (43.7 %)	24 (45.3 %)/29 (54.7 %)	0.16
Body mass index (kg/m^2^)	23.3	23.5	0.91
Co-morbidity (no/yes)	91 (60.3 %)/60 (39.7 %)	18 (40.0 %)/35 (60.0 %)	<0.01
Hypertension	20	13	
Diabetes mellitus	12	3	
Respiratory disease	6	3	
Heart disease	6	6	
Renal disease	5	3	
Brain infarction	5	4	
Connective tissue disease	3	2	
Liver disease	3	1	
Performance status (0, 1/2, 3)	137 (90.7 %)/14 (9.3 %)	38 (71.7 %)/15 (28.3 %)	<0.01
Location (C/A/T/D/S/R)	8 (5.3 %)/18 (11.9 %)/10 (6.6 %)/8 (5.3 %)/34 (22.5 %)/73 (48.4 %)	4 (7.5 %)/12 (22.6 %)/5 (9.4 %)/3 (5.7 %)/12 (22.6 %)/17 (32.2 %)	0.28
Tumor type (0/1/2/3/4/5)	17 (11.3 %)/25 (16.6 %)/94 (62.2 %)/13 (8.7 %)/1 (0.6 %)/1 (0.6 %)	1 (1.9 %)/7 (13.2 %)/38 (71.7 %)/6 (11.3 %)/0/1 (1.9 %)	0.26
Tumor size (mm)	69 (9–103)	47 (9–87)	0.36
Tumor depth (m/sm/mp/ss/se/ai)	0/2 (1.3 %)/19 (12.6 %)/108 (71.5 %)/12 (7.9 %)/10 (6.7 %)	0/2 (3.8 %)/4 (7.5 %)/37 (69.8 %)/7 (13.2 %)/3 (5.7 %)	0.74
Lymph node metastasis (N1/2/3)	98 (64.9 %)/38 (25.2 %)/15 (9.9 %)	34 (64.2 %)/13 (24.5 %)/6 (11.3 %)	0.95
CEA	8.1	9.1	0.69
CA19-9	26.1	21.4	0.97

### Surgical features and outcomes

Lymphatic invasion was significantly more frequent in EP than in YP (*p* = 0.02). No significant differences in histological type, vessel invasion, operation time, blood loss, operative procedures, or combined resection were seen between groups. Postoperative complications and length of hospital stay likewise did not differ between groups. Among YP, 76 % (115/151) had received AC, compared to only 40 % (21/52) among EP (*p* < 0.01; Table 2).

**Table 2:** Relationship between tumor and surgical features and outcomes

	Age <75 years	Age ≥75 years	*p*
Histological grade (well/mod/poor)	54 (35.8 %)/83 (55.0 %)/14 (9.2 %)	19 (35.8 %)/28 (52.8 %)/6 (11.4 %)	0.99
Lymphatic invasion (no/yes)	6 (4.0 %)/145 (96.0 %)	6 (11.3 %)/47 (88.7 %)	0.02
Vessel invasion (no/yes)	20 (13.2 %)/131 (86.8 %)	7 (13.2 %)/46 (86.8 %)	0.99
Operation time (min)	480 (80–713)	241 (74–645)	0.35
Blood loss (g)	185 (10–1400)	129 (20–510)	0.07
Laparoscopic surgery (no/yes)	83 (55.0 %)/69 (45.0 %)	31 (58.5 %)/22 (41.5 %)	0.62
Composite resection (no/yes)	143 (94.7 %)/8 (5.3 %)	48 (90.6 %)/5 (9.4 %)	0.38
Postoperative chemotherapy (no/yes)	36 (23.8 %)/115 (76.2 %)	32 (60.4 %)/21 (39.6 %)	<0.01
Postoperative complication (no/yes)	95 (62.9 %)/56 (37.1 %)	32 (60.4 %)/21 (39.6 %)	0.74
Hospital stay (days)	25.7 (14–31)	25.5 (16–40)	0.93

### Types of AC

Among YP who received AC, 62 % (71/115) received single-agent chemotherapy (TS-1, *n* = 29; tegafur-uracil, *n* = 33; capecitabine, *n* = 9) and 38 % (44/115) received combination therapy (FOLFOX, *n* = 19; SOX, *n* = 14; XELOX, *n* = 11). On the other hand, among EP, 71 % (15/21) were administered single-agent chemotherapy (TS-1, *n* = 3; tegafur-uracil, *n* = 11; capecitabine, *n* = 1) and only 29 % (6/21) received combination therapy (FOLFOX, *n* = 4; SOX, *n* = 1; XELOX, *n* = 1). Significant differences were evident between groups in the selection of chemotherapeutic agents (*p* < 0.01) (Table 3).

**Table 3:** Types of postoperative chemotherapy

	Age <75 years	Age ≥75 y	*p* value
*n*	115	21	
Single agent	71	15	<0.01
Combination therapy	44	6	
FOLFOX	19	4	
SOX	14	1	
XELOX	11	1	
TS-1	29	3	
UFT	33	11	
Capecitabine	9	1	

*UFT* 5-fluorouracil, oxaliplatin, *FOLFOX* 5-fluorouracil, and folinic acid, *SOX* S-1 and oxaliplatin, *XELOX* capecitabine plus oxaliplatin

### Tolerability of chemotherapy

Thirteen of the 71 YP (18.3 %) who received single-agent chemotherapy experienced side effects of grade 3 or greater, compared to 19 of 44 YP patients (43.2 %) who received combination therapy (median follow-up period, 35 vs. 51 months, respectively). On the other hand, none of the EP who received single-agent chemotherapy experienced severe side effects (0 %), compared to EP (33.3 %) who received combination therapy. In both groups, neutropenia was the most frequent side effect in this study. In YP, AC with single/combination agents was discontinued in eight of 71 patients (11.3 %) and six of 44 patients (13.6 %), respectively. On the other hand, in EP, AC with single/combination agents was discontinued in one of 15 patients (6.7 %) and none of six patients (0 %) (Table 4). During the observation period, one patient in the YP group who received mFOLFOX6 died due to acute pneumonia.

**Table 4:** Side effects and incidence of discontinuation of therapy in patients who received adjuvant chemotherapy

		Age <75 years		Age >75 years	
		Single	Combination	Single	Combination
*n*		71	44	15	6
Side effects ≥grade 3		13 (18.3 %)	19 (43.1 %)	0 (0 %)	2 (33.3 %)
Signs and symptoms					
Neutropenia		3	9	0	2
Anorexia		3	3	0	0
Diarrhea		3	2	0	0
Pneumonia		0	1	0	0
Anaphylaxis		0	1	0	0
General fatigue		0	1	0	0
Perforation		0	1	0	0
Acute leukoencephalopathy		0	1	0	0
Liver dysfunction		1	0	0	0
Hand-foot syndrome		1	0	0	0
Hyperbilirubinemia		2	0	0	0
Discontinuation		8 (11.3 %)	6 (13.6 %)	1 (6.7 %)	0 (0 %)

### Kaplan-Meier curves of the effect of chemotherapy on disease-free and overall survival

**Fig. 1:**
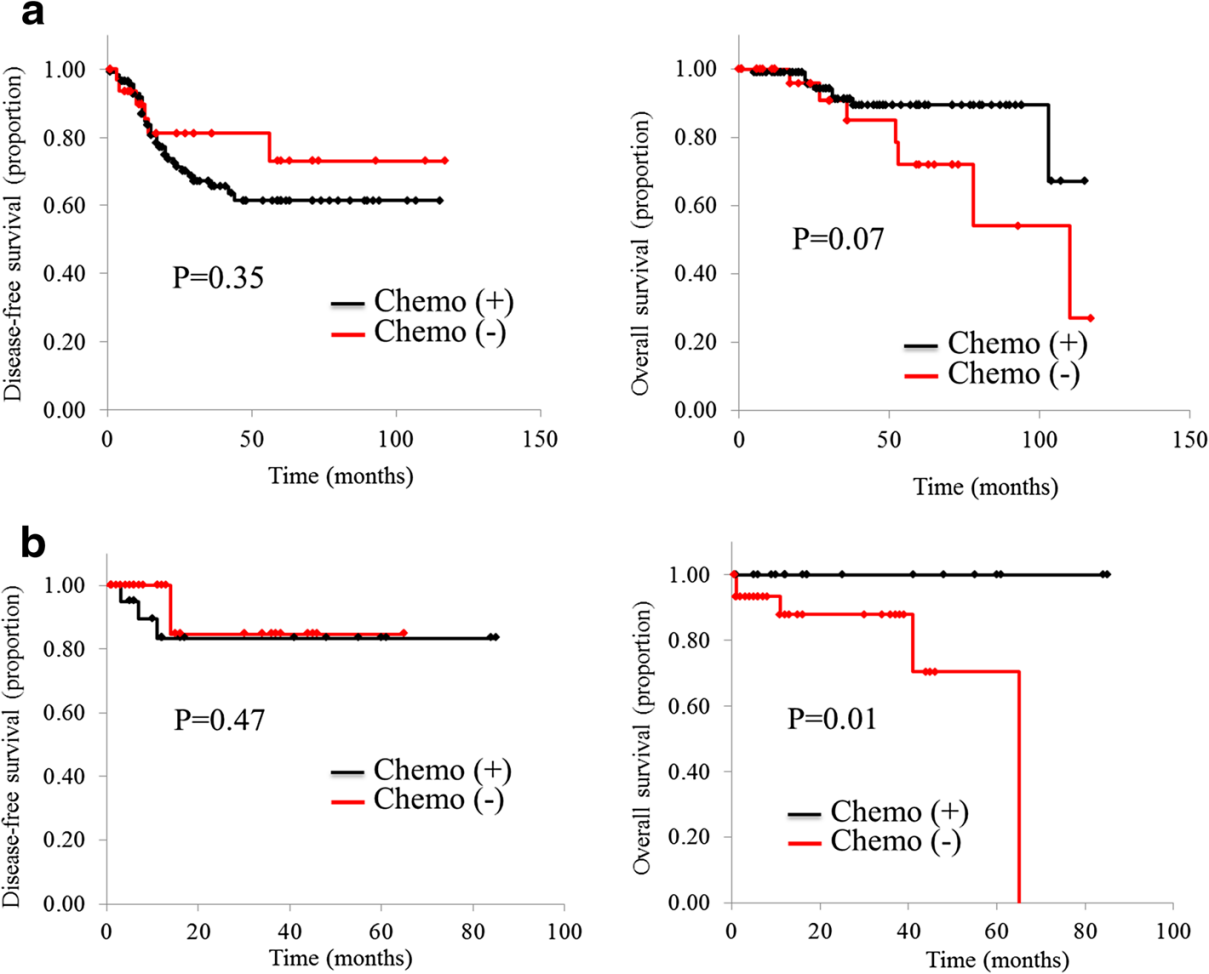
Disease-free survival and overall survival of stage III colorectal cancer patients. The subgroup of patients who received adjuvant chemotherapy displayed improved survival outcomes for both younger patients (*p* = 0.07; **a**) and elderly patients (*p* = 0.01; **b**)

YP showed no significant differences in disease-free survival between chemotherapy and non-chemotherapy subgroups (*p* = 0.35), and while overall survival tended to be better in the chemotherapy subgroup, the effect was still not significant (*p* = 0.07) (Fig. 1a). Likewise in EP, no significant difference in disease-free survival was evident between chemotherapy subgroups (*p* = 0.47). However, overall survival was significantly better in the chemotherapy subgroup (*p* = 0.01) (Fig. 1b).

## Discussion

Although the proportion of patients receiving AC was much lower among EP than among YP, the present results suggest that postoperative AC might be effective for improving overall survival in EP. In addition, the entire course of AC was able to be completed in many EP without severe side effects.

Previous studies have revealed the survival benefits of postoperative AC for patients with stage III colorectal cancer [16–18]. However, emphasis has been placed on careful diligence in assessing the indications for AC in EP, since these patients often show low performance status, major organ dysfunction, and high frequencies of comorbidities [19, 20]. In the present study, the frequency of comorbidities was higher and performance status was lower in EP than in YP (*p* < 0.01 each).

In the 1990s, the rate of administering AC was reportedly lower in EP (10–32 %) than in YP (55–77 %) [9, 10]. Ko and colleagues recently examined data from 810 colorectal cancer patients with lymph node metastasis [1]. They revealed that AC tended to be administered less frequently to EP (57 %) than to YP (91 %), as previously reported, even though the benefits of AC have been widely recognized. Furthermore, combination therapy was selected less often for EP (32 %) than for YP (74 %). In our study, the proportion of patients receiving AC was lower among EP (21/52, 40 %) than among YP (115/151, 76 %). In addition, the frequency of administering combination therapy was significantly lower in EP (28.6 %) than in YP (38.2 %, *p* < 0.01). The most common reason for withholding AC from EP was identified as patient age (15/32; 47 %) (Additional file 1: Table S1). In the YP group, AC was most often withheld from patients due to patient choice (15/36; 42 %). Such findings are supported by the results of a previous report on reasons for withholding AC [21].

Some studies have revealed that EP are at increased risk of developing side effects, such as nausea, stomatitis, vomiting, and neutropenia [9, 22–25]. However, other investigations have indicated that the rate of side effects does not differ between EP and YP [26, 27]. In a randomized trial involving 1014 patients, incidences of neutropenia, gastrointestinal toxic effects, and dermatitis were not significantly different between different age groups [28]. In our study, the rate of AC completion was high in the EP group, and the incidence of side effects of grade 3 or greater was lower in EP than in YP. Although our study may have involved some degree of selection bias, since we made deliberate choices in terms of performance status, general status, and cognitive function, particularly in EP, our results suggest that EP can safely receive postoperative AC if the selection of therapy is appropriate.

Steinberg and colleagues examined 1296 colorectal cancer patients with local invasion or positive lymph nodes [26]. They compared groups with and without AC and found significantly lower cancer recurrence and overall death rates in the AC group. Other studies have likewise revealed benefits in terms of disease-free survival and overall survival from the use of postoperative AC [27–30]. In the present study, in both YP and EP, overall survival was better among patients who received AC than among those who only underwent surgery (*p* = 0.07 and *p* = 0.01, respectively). Sanoff and colleagues examined the effects of AC in 5489 colorectal cancer patients 75 years of age and older [31]. They reported that the incidence of AC administration declined with patient age. They also indicated that EP could gain survival benefits from AC (hazard ratio, 0.60; 95 % CI, 0.53–0.68), supporting the present results. Other randomized clinical trials have also revealed that AC offers improved disease-free and overall survival in colorectal cancer patients over 70 years of age [32–34]. About 40 % of colorectal cancer patients are reportedly over 75 years old [35], yet older patients remain underrepresented in clinical trials of chemotherapy because of the scarcity of efficacy data [32]. In the future, steps should be taken to ensure greater inclusion of older patients in such clinical trials.

Chronological age reportedly does not correlate with physiological age [36–39]. However, many older people are not provided AC because of their advanced chronological age (Additional file 1: Table S1) [1]. Several geriatric assessment tools that could help predict patient outcomes are now available [40–42]. Hurria and colleagues also revealed a predictive model of chemotherapy toxicity for older patients, and application of such tools should be considered when selecting chemotherapies for older patients [43]. A previous report revealed that 29 % of colorectal cancer patients older than 70 years died due to disease recurrence, while 13 % died due to causes unrelated to recurrence [9]. These results indicate that appropriate chemotherapy in older patients might confer a survival benefit in terms of cancer control, and clinicians should thus not hesitate to aggressively treat cancers in EP, just as in YP.

The main drawback of this study was the selection bias, in that only patients fit for chemotherapy were administered treatment. Thus, definition of the circumstances in which chemotherapy may be selected for node-positive patients who are elderly is paramount. A randomized controlled trial is more likely to yield an adequate assessment of whether EP should be administered AC under strict selection criteria.

## Conclusions

The present findings suggest that postoperative AC could be effective in improving overall survival following resection of stage III colorectal cancer, not only in YP but also in EP. AC should therefore not be withheld from eligible EP purely on the basis of advanced age.

## Abbreviations

AC, adjuvant chemotherapy; NAC, neoadjuvant chemotherapy

## Additional file

Additional file 1: Table S1. Reasons for withholding adjuvant chemotherapy. (DOC 46 kb)
