# Supplementary material for: Effectiveness of adjuvant chemotherapy for elderly patients with lymph node-positive colorectal cancer
Source: World J Surg Oncol. 2016 Jul 28;14:197. doi: 10.1186/s12957-016-0959-5 (PMC4964014; doi:10.1186/s12957-016-0959-5)
Supplement: Additional file 1: Table S1. — Reasons for withholding adjuvant chemotherapy. (DOC 46 kb) [file 12957_2016_959_MOESM1_ESM.doc]

**Supplementary Table 1.** Reasons for withholding adjuvant chemotherapy

|  | **Age < 75 y** | **Age ≥ 75 y** |
| --- | --- | --- |
| **n** | **151** | **53** |
| **Number of patients who withheld adjuvant chemotherapy** | **36 (24%)** | **32 (60%)** |
| **Patient age** | **0** | **15 (47%)** |
| **Co-morbidity** | **12 (33%)** | **7 (21%)** |
| **Low performance status** | **5 (14%)** | **5 (16%)** |
| **Patient choice** | **15 (42%)** | **5 (16%)** |
| **Reason unknown** | **4 (11%)** | **0** |
